# Comparative study between Levobupivacaine and Bupivacaine for hernia surgery in the elderly

**DOI:** 10.1186/1471-2482-12-S1-S12

**Published:** 2012-11-15

**Authors:** Rita Compagna, Gabriele Vigliotti, Guido Coretti, Maurizio Amato, Giovanni Aprea, Alessandro Puzziello, Carmelo Militello, Fabrizio Iacono, Domenico Prezioso, Bruno Amato

**Affiliations:** 1 Endocrinosurgery Unit, Dept of Medical and Surgical Sciences, University Magna Graecia, Italy Catanzaro, Italy; 2 Department of Surgical and Gastroenterological Sciences, University of Padua, Italy; 3 Department of Urology, School of Medicine, University “Federico II” of Naples, Italy; 4 Department of General, Geriatric, Oncologic Surgery and advanced technologies, University "Federico II" of Naples, Via Pansini 5 - 80131 - Naples, Italy

## Abstract

**Background:**

The inguinal hernia is one of the most common diseases in the elderly. Treatment of this type of pathology is exclusively surgical and relies almost always on the use of local anesthesia. While in the past hernia surgery was carried out mainly by general anesthesia, in recent years there has been growing emphasis on the role of local anesthesia.

**Methods:**

The aim of our study was to compare intra-and postoperative analgesia obtained by the use of levobupivacaine compared with that of bupivacaine. Bupivacaine is one of the main local anesthetics used in the intervention of inguinal hernioplasty. Levobupivacaine is an enantiomer of racemic bupivacaine with less cardiotoxicity and neurotoxicity. The study was conducted from April 2010 to May 2012. We collected data of forty male patients, aged between 73 and 85 years, who underwent inguinal hernioplasty with local anesthesia for the first time.

**Results:**

Minimal pain is the same in both groups. Mild pain was more frequent in the group who used bupivacaine, moderate pain was slightly more frequent in the group who used levobupivacaine, and the same for intense pain. It is therefore evident how Bupivacaine is slightly less preferred after four and twenty four hours, while the two drugs seem to have the same effect at a distance of twelve and forty-eight hours. Bupivacaine shows a significantly higher number of complications, as already demonstrated by previous studies. The request for an analgesic was slightly higher in patients receiving levobupivacaine.

**Conclusions:**

After considering all these elements, we can conclude that the clinical efficacy of levobupivacaine and racemic bupivacaine are essentially similar, when used under local intervention of inguinal hernioplasty.

## Background

The inguinal hernia is one of the most common diseases in the elderly. The Italian National Health System is geared to recognize the role of local anesthesia for the surgical treatment of inguinal hernia. Treatment of this type of pathology is exclusively surgical and relies almost always on the contribution of local anesthesia. While in the past, hernia surgery was carried out mainly by general anesthesia, in recent years there has been growing emphasis on the role of local anesthesia. This type of anesthesia has significantly improved the treatment of inguinal hernia, significantly reducing recurrences, complications, recovery time and return to normal working activities.

Hernia surgery should be approached according to a technique as simple and safe as possible that is at the same time accepted by the patient and easily realizable by the surgeon [1]. Inguinal hernioplasty is now the most performed surgery in the departments of general surgery [2]. For this reason it is necessary to find solutions which can be adapted to each individual case, combining experience and innovation. Surgery can be customized according to physique, age, comorbidity, lifestyle and size of the hernia. We are talking more and more about Tailored Surgery, the so-called personalized surgery, individualized, built on the needs and characteristics of the patient [3]. The concept of Tailored Surgery encompasses not only technical-surgical and prosthetic choices but also anesthetic (assisted local, spinal or loco-regional, general). According to recent guidelines of the European Hernia Society, published in “Hernia” in 2009, the repair of a hernia in primary election can always take advantage of local anesthesia. This is a grade A recommendation, with high scientific impact [4]. The simultaneous use of local anesthetic drugs with a long duration of action, but very powerful such as Levobupivacaine (Chirocaine), in addition to drugs equally potent, but duration of immediate action, such as Mepivacaine (Carbocaine), allow optimization of anesthesia / analgesia both intra-and post-operatively. Finally, do not forget that we are talking about local assisted anesthesia and therefore the contribution of the anesthetist, and the overall effectiveness of the anesthesia, are essential to ensure the maximum comfort to the patient intraoperatively [5]. Why does the surgeon have to practice this kind of anesthesia? Because this type of anesthesia consists of several phases: the first, percutaneous, may be made without distinction by the surgeon or anesthesiologist, while the last phase, incisional, is exclusively of surgical pertinence, as it is the task of the surgeon to identify the points of landmarks, locate and infiltrate properly. Local Assisted Anesthesia by truncal block / incisional has several advantages: safety, even in patients at risk; effectiveness, commitment to anesthetic proportionate intervention, minimally invasive anesthetic technique, simple and reproducible. Currently local assisted anesthesia is the procedure of choice in primary unilateral inguinal hernias treated in election. There are no absolute contraindications to the anesthetic block. If anything, there are relative contraindications: poor patient, especially at a young age, morbid obesity, bilateral hernioplasty, bulky inguinal hernias [6].

The aim of this study was to compare two local anesthetics, levobupivacaine and bupivacaine, commonly used in the surgical treatment of inguinal hernia.

## Methods

From April 2010 to May 2012 we studied forty patients recovered in the department of General Surgery, University of Naples “Federico II”, affected by inguinal hernia for the first time and treated in this period of time. The patients were divided into two groups, corresponding to the two drugs that we have studied. We interviewed patients at the end of the operation using the VAS Scale. During the interview, we have focused on some aspects: the intra-operative pain, post-operative pain, need for analgesia in the postoperative period and the overall satisfaction with anesthesia. We compared the results obtained and we made interesting observations, for a condition which, we are sure, will be increasingly common in the coming years due to progressive aging of the Italian population. We collected data of forty male patients, aged between 65 and 85 years, who underwent inguinal hernioplasty under local anesthesia for the first time. In Table 1 we reported the main characteristics of patients. Patients were divided into two groups using a double-blind randomized system. The first group (A) received Levobupivacaine (n = 20), the second (B) received bupivacaine (n = 20). During surgery, the patients were continuously monitored with ECG intraoperative and pulse oximeter. In the first group A twelve patients were treated for direct hernia and eight patients for indirect hernia. In the second group B eleven patients were treated for direct hernia and nine patients for indirect hernia. In Levobupivacaine group, the mean operative time was 43 minutes. While in the bupivacaine group the mean operative time was 40 minutes exactly. In group A average time anesthesiological was sixty minutes. In group B the time was fifty minutes for bupivacaine. The amount of fentanyl used was respectively 115 mcg in the first group of interventions and 119 mcg in the second group. The location of the operation in the levobupivacaine group, the ratio right / left was twelve to eight; in the bupivacaine group this ratio was fifteen to five. Finally we reported the ASA scale: ten patients of the first group were classified in stage I and ten patients in stage II. In the second group eight patients were classified in stage I and twelve patients in stage II. No patients in stage III. The anesthetic block was made employing the following protocol: the first phase, percutaneous, allowed us to obtain a block on the troncular selective ilioinguinal and iliohypogastric nerves through a puncture performed two cm medial to the anterior superior iliac spine, lateral to the rectus muscle of abdomen. For this purpose we used 7-8 cc of Levobupivacaine (or Bupivacaine) at 7.5%. The second phase, percutaneous, blocked the genital branch of the genitofemoral nerve, through a puncture performed below the inguinal ligament, lateral to the pubic tubercle. We used 2-3 cc of levobupivacaine (or Bupivacaine) at 7.5%, a very powerful local anesthetic was used with a long duration of action, to ensure good analgesia both intra-and post-operatively. The third phase, percutaneous, was completed by infiltration of the surgical incision using a 22 gauge spinal needle employing 10 or 15 cc of Mepivacaine hydrochloride at 2%. The anesthetic block was completed in the incisional phase by means of an open air infiltration, performed for each anatomical floor in the course of surgery, using the Mepivacaine hydrochloride at 2%. The points requiring infiltration were four: the end of the external oblique muscle (8, 10 cc), the pubic tubercle (2 cc), the medial and lateral pillar external inguinal orifice (2, 3 cc), the orifice internal inguinal (2 3 cc) ; other locations in case of need or in large hernias can be: funiculus in the sub-cremasterica; genitofemoral nerve in the sub-cremasterica and the hernial sac. With regard to the surgical techniques, in patients with direct inguinal hernia we realized the inguinal Lichtenstein hernioplasty. In patients with indirect inguinal hernia instead we used Rutkow and Robbins hernioplasty. Immediately after the operation, patients were interviewed to determine the extent of intra-and post-operative pain and the degree of satisfaction with surgery performed under local anesthesia. We used the VAS scale. The VAS scale is a straight line with two ends corresponding to "no pain" and the worst possible pain (or the maximum that he experienced). It is a one-dimensional tool that quantifies what patients subjectively perceive as pain or as a relief in all their physical, psychological and spiritual variables without distinguishing which of these components plays a greater role. This scale presents many important characteristics: it has the advantage of being simple, is easily understood by most patients, can easily be repeated and is particularly useful for monitoring the acute course.

**Table 1:** Patients characteristics

Parameters	Levobupivacaine	Bupivacaine	P value
Age	75(85-65)	75(87-73)	0,82 (*)
Sex(M/F)	20/0	20/0	
Weight(kg)	72	76	0,34 (+)
Direct hernias	12	11	0,51 (+)
Indirect hernias	8	9	
ASA status			0,65 (+)
1	10(50%)	8(40%)	
2	10(50%)	12(60%)	
3	0(0)	0(0)	
Fentanyl used (mcg)	115 +/- 25	119 +/- 33	0,33 (*)
Site			0,52 (+)
Right	12(60%)	15(75%)	
Left	8(40%)	5(25%)	
Operating time (min)	43(53-33)	40(53-27)	0,24 (*)
Anaesthesia time (min)	60(55-65)	50(45-55)	0,28 (*)

Test Used

(+) Chi square

(*) ANOVA

## Results

In Table 2 we show data related to the intra-operative pain. In the group of patients who received a local levobupivacaine anesthetic, we identified two with minimal pain, eleven mild pain, six moderate pain and one intense pain. In the group of patients who received bupivacaine, two experienced minimal pain, thirteen mild, five with moderate pain and no one intense pain. We then we focused on post-operative pain. The degree of post-operative pain is generally recognized in three positions: in the supine position, in the passage from the supine position to sitting, and during a short walk. In Table 3 we reported the results of post-operative pain. In the levobupivacaine group two patients experienced pain in the supine position, three seated and two standing. In the Bupivacaine group, two patients identified pain in the supine position, three seated and three standing. Then we collected the impressions of patients even after several hours from surgery. Four hours after the operation, three patients in the first group identified pain and two patients in the second group identified pain. Twelve hours after surgery, two patients in the first group and two in the second identified pain. Twenty-four hours after surgery, two patients in the first group and one in the second identified pain. Forty-eight hours after surgery, a patient in the first group and one patient in the second group identified pain. In the postoperative period patients received paracetamol as an analgesic drug up to three times a day, depending on the need. In Table 4 we evaluated two important elements: complications and overall satisfaction with the intervention. With regard to complications in the group of Levobupivacaine, four patients experienced nausea / vomiting, one itching, and no one infection. Instead in the bupivacaine group, five patients experienced nausea / vomiting, one itching and one infection. The overall satisfaction was assessed using a scale of three levels. In group A of levobupivacaine, thirteen patients said they were fully satisfied, six moderately satisfied and only one just satisfied. In the second group, thirteen patients were fully satisfied and seven were moderately satisfied while no one said he was just satisfied. In Table 5 we collected the data on the need of analgesic in the immediate post-operative phase. The patients of the first group who required paracetamol were 14. Instead, we administered paracetamol in twelve patients of the second group. In the levobupivacaine group the average time to first request of paracetamol was approximately 226 minutes (about four hours), in the bupivacaine group was about 367 minutes (approximately six hours). The number of patients of the first group who required other analgesics for pain relief within twenty-four hours was six. Five patients of the second group required others analgesics.

**Table 2:** Intra-operative Pain

Type of pain	Levobupivacaine	Bupivacaine	*P value*
Minimal	2	2	0,28
Mild	11	13	
Moderate	6	5	
Intense	1	0	

**Table 3:** Post-operative Pain

Position	Levobupivacaine	Bupivacaine	*P value*
Supine	2	2	*0*,*7*
Sitting	3	3	*0*,*98*
Standing	2	3	*0*,*27*
Time			
4 h	3	2	*0*,*76*
12 h	2	2	*0*,*41*
24 h	2	1	*0*,*09*
48 h	1	1	*0*,*25*

**Table 4:** Post-operative complications and patient satisfaction

Complications	Levobupivacaine	Bupivacaine	*P value*
Nausea/Vomiting	4	5	0,67
Itching	1	1	
Infection	0	1	
Patient satisfaction			
Completely satisfied	13	13	0,71
Moderately satisfied	6	7	
Satisfied	1	0	

**Table 5:** Intake of analgesics in post-operative period

Parameters	Levobupivacaine	Bupivacaine	*P value*
Time of first request of paracetamol (min)	226	367	*P*=*0*,*141*
Request of paracetamol in 24 hours (N.patients)	14	12	*P*=*0*,*852*
Need for other analgesics (N. patients)	6	5	

Test Used

Chi Square

### Statistical analysis

In this study, continuous variables was reported as an average, more or less the standard deviation, and analyzed using ANOVA (analysis of variance). It is a parametric test that is used in statistics to compute the variance between two or more different groups. Analysis of variance is a set of statistical techniques that are part of the inferential statistics that allow us to compare two or more groups of data comparing the internal variability of these groups with the variability between groups. Categorical variables were reported as proportions instead and analyzed using chi-square test. Chi-square test is one of the tests used in statistics using the chi-square variable causal to verify if the null hypothesis is probabilistically compatible with the data. The values relating to the intra-operative pain and post-operative pain, as well as those relating to the taking of analgesics during the postoperative course, were always reported and analyzed through chi-square test. A P value less than 0.05 was considered statistically significant. Based on previous studies, the difference in the level of pain between the group of levobupivacaine and bupivacaine was 1.5.

## Discussion

International literature shows how local anesthesia is certainly more advantageous in terms of costs for the structure. While there are no particular differences between regional and general anesthesia, local anesthesia results seem to be better. A potential advantage of local anesthesia realized without any monitoring or additional drugs administered intravenously (the so-called local anesthesia not monitored) [7].

Levobupivacaine is a local anesthetic with long duration of action. It works by blocking nerve conduction of sensory and motor nerves, interacting predominantly with the voltage-gated sodium channels in the membrane of the cell, but also blocking potassium channels and calcium. Levobupivacaine also interferes with the transmission of the pulse and the conduction in other tissues where the effects on the central nervous system and cardiovascular system are the most important for the occurrence of clinical adverse reactions. Chirocaine is a compound based levobupivacaine hydrochloride. It is capable of producing a block on both the sympathetic system and on the parasympathetic system demonstrating hemodynamic changes significantly milder than Ropivacaine, which instead has the greatest influence on the sympathetic system with respect to that parasympathetic [8]. The dose of levobupivacaine is expressed as a basis, unlike the racemic Bupivacaine where the dose is expressed as a hydrochloride salt. This roughly translates into a 13% more active ingredient in the solutions of levobupivacaine compared to those of bupivacaine. As regards to the pharmacokinetic properties, in human trials, the kinetics of distribution of levobupivacaine after intravenous administration are essentially the same as bupivacaine. The plasma concentration of levobupivacaine following therapeutic administration depends on the dose and, as absorption from the site of administration is influenced by the vascularity of the tissue, the route of administration. It is available in two formulations: Vial of 10 ml polypropylene, in pack sizes of 5, 10 and 20 units, polipropilene vial of 10 ml in sterile blister packs of 5, 10 and 20 units. Chirocaine can be worked in a very large number of surgical procedures, can be administered in major surgery for epidural, intrathecal, in nerve conduction block device, in minor surgery for local infiltration and for ophthalmic use in order to obtain a peribulbar block. It could be used in the treatment of pain, as an analgesic in the course of delivery, both for bolus infusion, and also for the post-operative pain. Among the uses of Chirocaine there are scientifically proven mastopexy interventions [9]. Levobupivacaine is more effective to obtain analgesia with local infiltration compared to Ropivacaine, providing analgesia for postoperative period. Interventions of septoplasty and rhinoseptoplasty with an infiltration of levobupivacaine at 0.25% in the nasal region improve the post-operative analgesia and reduce the demand for additional analgesia during the twenty-four hours following nasal surgery. The post-operative analgesia achieved through the local infiltration of levobupivacaine has been demonstrated to be significantly more powerful and showed longer duration compared to the association lidocaine plus epinephrine. The same holds with regard to the interventions of mini-abdominoplasty [5]. In this case levobupivacaine has proved to be more effective and with a duration indeed higher than ropivacaine. Levobupivacaine can be the agent of first choice in the thoracic epidural block [10] , compared to the use of a Ropivacaine dose equivalent. It has also proved effective even in the interventions of arthroscopy and Carotid Endarterectomy [11].

Especially in recent years local anesthesia allows the surgeon to monitor patients and to have simultaneously shorter hospitalization times and lower costs for the structure. Local anesthesia applied during endarterectomy surgery allows the surgeon to assess the levels of cerebral perfusion in an awake patient, giving a better chance of cerebral protection during arterial clamping. All these elements indicate that such interventions performed under local anesthesia with levobupivacaine compounds offer greater chances of success with significantly reduced rates of morbidity and mortality [12-14].

Locally hernioplasty has proved to be the method with the minor impact on the functioning of organs and systems, as it appears to be safe, effective, with a low incidence of side effects, enabling a rapid mobilization of the patient and significantly reducing the time of hospitalization, in less than twenty-four hours [15].

Among rare complications of surgery, hernioplasty under local anesthesia include: cardiovascular instability, nausea, vomiting, urinary retention, scrotal hematoma, edema, infection, orchitis, testicular atrophy and recurrences. Normally this type of surgery shows a lower incidence of complications than the same operation performed with general anesthesia. Compared with other types of anesthesia, post-operative complications of the respiratory and circulatory systems are significantly reduced [16].

The use of local anesthesia also allows the patient to be awake, aware, and thus able to collaborate actively conducting a stress-test by performing the Valsalva maneuver or a cough, which allows the surgeon to evaluate intra-operatively the presence of defects, latent trusses and sealing of the repair of plastic, reducing significantly the proportion of surgical failures [17,18].

The anesthetic block consists of four phases: The first phase, percutaneous, provides the block troncular selective ilioinguinal nerves and iliohypogastric. The second phase, percutaneous, blocks the genital branch of the genitofemoral nerve, through a puncture performed below the inguinal ligament, lateral to the pubic tubercle. The third phase, percutaneous, provides for the infiltration of the surgical incision using a 22 gauge spinal needle. The anesthetic block is completed in the incisional phase by means of an open infiltration performed in each anatomical floor during the course of surgery [19]. Local anesthesia with levobupivacaine and bupivacaine is now a established and safe procedure with risks considerably reduced, a quick and full recovery of the patient's general condition and an immediate return to normal working activities. Data from the international literature indicate how the levobupivacaine is less toxic compared to bupivacaine, both at the cardiac level and at the neurological level [20,21].

The purpose of this study was to compare the perception of pain intra and post-operative, found as a result of intervention with the Levobupivacaine, compared to that recorded after the same intervention carried out with the racemic bupivacaine. We used the same dose for both anesthetics. The forty patients we studied, were randomly distributed in two groups, and were classified on the basis of a number of variables: age, weight, sex, type of hernia, ASA Stadium and location of the hernia. The first point on which we focused was intra-operative pain. In the group of patients treated with levobupivacaine, 10 % reported minimal pain, 55% mild pain, 30% moderate pain, 5% severe pain. In the group of patients treated with bupivacaine, 10% identified minimal pain, 65% mild pain, 25% moderate pain and no one intense pain. Therefore, we can say that minimal pain is the same in both groups. Mild pain was more frequent in the bupivacaine group, moderate pain slightly more frequent in the Levobupivacaine group and the same for intense pain. The second point on which we focused was post-operative pain, assessed in three positions within 48 hours. In the first group, 10% of patients reported pain in the supine position, 15% in the sitting position and 10% standing up. In the second group, 10 % reported pain in the supine position, 15% in the sitting position and 15% standing up. Therefore the data show the same results for the first two positions and a slight preference for levobupivacaine in the upright position. With regard to the assessment of pain during the forty-eight hours, we evaluated the impressions of the patient's at four time intervals: four, twelve, twenty-four and forty-eight hours. In the levobupivacaine group, 15% of patients expressed pain relief after four hours, 10% after 24 hours, 5% after 48 hours. In the bupivacaine group, 10% of patients experienced pain after four and twelve hours, 5% after twenty-four and forty-eight hours. It is therefore evident how Bupivacaine is preferred slightly after four and twenty four hours, while the two drugs appear to be equivalent at a distance of twelve and forty-eight hours. The third point we considered werethe postoperative complications and overall patient satisfaction. In the Levobupivacaine group, 20% experienced symptoms such as nausea and / or vomiting, 5% itching, no one with an infection. In the bupivacaine group, 25% noted nausea and / or vomiting, 5% itching, 5% infection. Bupivacaine shows a significantly higher number of complications, as already demonstrated by previous studies. The overall satisfaction towards the intervention was high in 65% of patients receiving levobupivacaine, moderate in 30% and sufficient in 5%. Instead patients who received bupivacaine expressed 65% complete satisfaction and 35% satisfaction moderate. In neither of the two groups were found signs of toxicity by local anesthetic, such as tinnitus, pallor circumorale, cardiovascular or neurological manifestations. Finally, the last point on which we have focused our work has been the application of analgesic in post-operative period. Seventy percent of patients who received levobupivacaine required at least an analgesic (paracetamol) within twenty-four hours surgery and 30% required others analgesics. In the bupivacaine group, 60% took some paracetamol after twenty-four hours, 25% required other analgesics. The request for ananalgesic was slightly higher in patients receiving levobupivacaine.

## Conclusions

After considering all these factors, we can conclude that the clinical efficacy of levobupivacaine and racemic bupivacaine are essentially similar. When we perform inguinal hernioplasty surgery with local anaesthesia, Levobupivacaine could be preferred because it has a lower cardiac and neurological toxicity compared to bupivacaine, as previously demonstrated by other clinical studies.

## Competing interests

The authors declare that they have no competing interests.

## Authors’ contributions

RC, BA: conception and design, interpretation of data, given final approval of the version to be published; GV, GC, MA, GA: acquisition of data, drafting the manuscript, given final approval of the version to be published; AP, CM, FI, DP: critical revision, given final approval of the version to be published.
